# A Genome Scan Conducted in a Multigenerational Pedigree with Convergent Strabismus Supports a Complex Genetic Determinism

**DOI:** 10.1371/journal.pone.0083574

**Published:** 2013-12-23

**Authors:** Anouk Georges, Nadine Cambisano, Naïma Ahariz, Latifa Karim, Michel Georges

**Affiliations:** 1 Department of Ophtalmology, Faculty of Medicine, University of Liège (CHU), Liège, Belgium; 2 Unit of Animal Genomics, GIGA-R & Faculty of Veterinary Medicine, University of Liège (B34), Liège, Belgium; 3 GIGA-R Genotranscriptomics Core Faclity, University of Liège (B34), Liège, Belgium; Justus-Liebig-University Giessen, Germany

## Abstract

A genome-wide linkage scan was conducted in a Northern-European multigenerational pedigree with nine of 40 related members affected with concomitant strabismus. Twenty-seven members of the pedigree including all affected individuals were genotyped using a SNP array interrogating > 300,000 common SNPs. We conducted parametric and non-parametric linkage analyses assuming segregation of an autosomal dominant mutation, yet allowing for incomplete penetrance and phenocopies. We detected two chromosome regions with near-suggestive evidence for linkage, respectively on chromosomes 8 and 18. The chromosome 8 linkage implied a penetrance of 0.80 and a rate of phenocopy of 0.11, while the chromosome 18 linkage implied a penetrance of 0.64 and a rate of phenocopy of 0. Our analysis excludes a simple genetic determinism of strabismus in this pedigree.

## Introduction

In Caucasians, an estimated 2–4% of the population suffers from non-syndromic misalignment of the eyes, also referred to as squint or strabismus. A heritable component of predisposition to strabismus had been suspected since antiquity, and is supported by (i) a higher concordancy rate amongst monozygotic (73%) than dizygotic (35%) twins, (ii) familial clustering (manifested as an increased in the incidence of strabismus in relatives of affected individuals), as well as (iii) markedly different incidences of strabismus between racial groups [1].

However, with the exception of Mendelian forms of incomitant strabismus including Congenital Fibrosis of the Extraocular Muscles (CFEOM) and Duane Retraction Syndrome (DRS), the molecular causes of strabismus remain largely unknown [1]–[3]. Linkage and association scans have been conducted for the more common concomitant forms of strabismus, yielding replicated evidence for only one risk locus (*STBMS1*) on chromosome 7p. However, the most likely mode of inheritance differed between the two reports, and *STBMS1* only accounted for the segregation of the condition in a minority of families [4]–[7]. Concomitant strabismus thus appears to have a complex, multifactorial determinism. Whether inherited predisposition to strabismus reflects the cumulative effect of common risk alleles that individually confer very modest increases in relative risk, or whether rare but highly penetrant alleles might account for at least part of familial clustering remains unknown.

## Results

**Figure 1:**
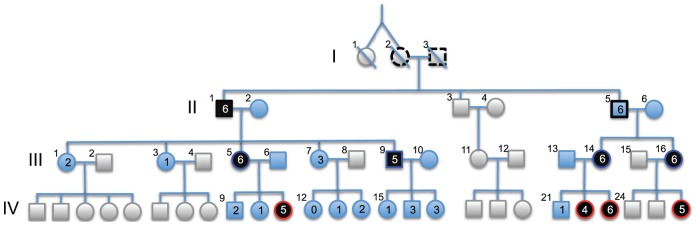
Multigenerational pedigree with convergent strabismus. Individuals for which DNA samples could be collected are represented in blue when unaffected and in black when affected. Assuming that all cases in the pedigree share an identical-by-descent (IBD) autosomal dominant mutation (i) one of the two grand-grand parents (individuals I.2 or I.3) has to be at least germ-line carrier as indicated by the black dotted lining, and (ii) grand-father II.5 is obligate carrier as indicated by the black lining. Under the same hypothesis, the parental origin of the mutation can be determined for all cases of generation III or IV as indicated by the red (maternal origin) or blue lining (paternal origin). The numbers within the symbols indicate the number of IBD risk haplotypes carried by the corresponding individual for the six putative loci, which are described in Table 1. The numbers outside the symbols are the individual identifiers within generation (roman numbers).

We identified a multigenerational pedigree with an unusually high incidence of convergent strabismus (Fig. 1). Eight of the 40 related members of the family were born with non-syndromic concomitant esotropy, while one developed similar symptoms at the age of five (III.9). The nine affected individuals underwent one or more surgical corrections before the age of six. The observed segregation pattern was compatible with autosomal dominant inheritance with incomplete penetrance: (i) both sexes were affected (two men, seven women), (ii) affected offspring generally had one affected parent, and (iii) the proportion of affected offspring from affected parents did not deviate significantly from 50% (6/17). Under this model, two asymptomatic individuals would be obligate carriers: (i) grand-father II.5 who transmitted strabismus to his two daughters, and (ii) one of the grand-grand-parents (I.2 or I.3) who transmitted the hypothetical mutation to two of their three sons. It is noteworthy that there was no recorded history of strabismus in other relatives of the grand-grand-parents (generation I). Taken together, these observations suggested that the high incidence of strabismus in this family might involve a highly penetrant, dominant mutation tracing back to one of the grand-grand-parents.

To test this hypothesis we collected samples of saliva from 27 of the family members with full consent, extracted genomic DNA, and performed genome-wide SNP genotyping using Illumina HumanCNV370-Quad HD Infinium arrays. We excluded 48,560/357,946 SNPs with call rate < 26/27 (96%). Analysis of the retained SNP genotypes confirmed all presumed familial relationships (data not shown).

The most parsimonious hypothesis predicts that an identical-by-descent (IBD) chromosome segment encompassing a dominant mutation, will be shared by the nine affected individuals and obligate carrier II.5, while it will not have been transmitted to any of the remaining unaffected relatives. Given the available samples, such hypothetical opposite segregation distortion (mutation transmitted by heterozygous parent to 9/9 cases, 1/1 obligate carrier and to 0/9 unaffected relatives) would yield genome-wide significant non-parametric and parametric p-values of 3.0×10^−5^ and 7.6×10^−5^ (corresponding to parametric and non-parametric lod scores of 3.8 and 3.4), respectively (see Methods). Thus, the studied pedigree provides adequate power to detect such highly penetrant dominant mutation if it exists. However, relatives other than II.5 may have inherited the mutation without being affected. Moreover, strabismus being a relatively common condition, some pedigree members may be affected without having inherited the hypothetical mutation that would explain the high incidence of strabismus in this family (whether due to distinct genetic or non-genetic factors). Thus, we allowed for (i) incomplete penetrance and (ii) phenocopies in our analyses.

**Figure 2:**
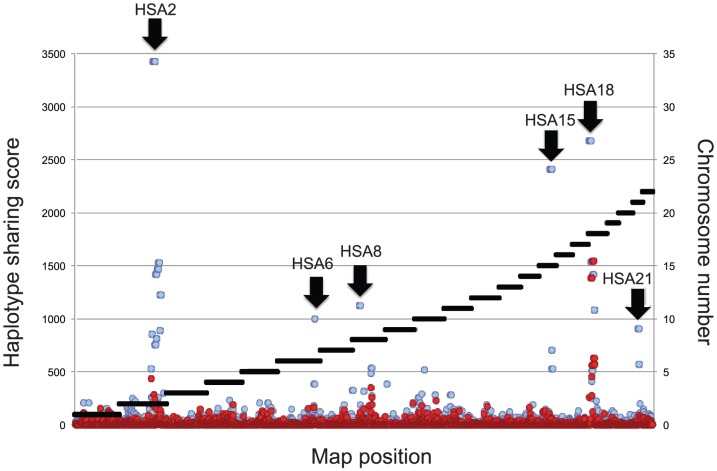
Haplotype sharing score across the genome. The limits between the 22 autosomes are indicated by the black horizontal bars (*right Y-axis*). The scores (*left Y-axis*) obtained when imposing haplotype sharing for the nine/nine cases plus the obligate carrier are given by the red circles. The scores obtained when imposing haplotype sharing for eight/nine cases plus the obligate carrier are given by the blue circles. The six identified chromosome segments are marked by the black arrows.

As the linkage signal is expected to accrue primarily from haplotype sharing amongst cases, we first scanned the genome for haplotypes shared by the nine cases and II.5 or at least eight cases and II.5 (to accommodate one phenocopy) using ASSDOM (see Methods). We detected one haplotype on chromosome 18 shared by the nine cases plus II.5, and five haplotypes shared by eight cases plus II.5, respectively on chromosomes 2, 6, 8, 15 and 21 (Fig. 2). It is noteworthy that the nine cases plus II.5 are expected to share on average 1.3 (95% confidence interval (CI): 0–4) IBD segments just by chance alone, while 8/9 cases plus II.5 are expected to fortuitously share 6.9 such segments (CI: 2–12)(see Methods). Consequently, these findings do not - on their own - provide adequate evidence for linkage. A haplotype shared by the nine cases plus II.5 has an expected size of 20 cM (CI: 2.4–56 cM), while one shared by 8/9 cases plus II.5 has expected size 22 cM (CI: 2.7–62.4cM) (see Methods). As can be seen from Table 1, shared segments were within the expected size range, although three approached the lower limit (HSA6, 8 and 21).

**Table 1:** Features of the six identified candidate loci.

**Chrom**	**N°**	**HSA2**	**HSA6**	**HSA8**	**HSA15**	**HSA18**	**HSA21**
	**Begin**	165,435,694	147,933,198	85,973,070	73,711,498	8,792,955	32,455,323
	**End**	219,809,031	151,679,962	89,157,125	86,981,023	27,191,190	36,595,076
	**Size**	54,373,337	3,746,764	3,184,055	13,269,525	18,398,235	4,139,753
	**Band**	2q24.3-2q35	6q24.3–6q25.1	8q21.2–8q21.3	15q24.2–15q25.3	18p11.22–18q12.1	21q22.11–21q22.12
**Recomb.**	**Prox**	II.2 or II.5	IV.26	II.2 or II.5	II.2 or II.5	IV.22	III.14
	**Dist**	III.9	II.2 or II.5	II.2 or II.5	IV.26	IV.11	IV.22
**Cases**	**T**	8	8	8	8	9	8
	**NT**	1	1	1	1	0	1
	**Phenocopy**	IV.22	IV.22	IV.26	III.9	-	IV.11
**Non-affected**	**T**	4	7	2	3	5	5
	**NT**	6	6	8	4	8	8
	**Non-expressing**	II.5IV.9-16-17	II.5, III.1-7IV.14-15-16-17	II.5III.1	II.5, III.3IV.21	II.5, III.7IV.13-14-16	II.5, III.7IV.9-10-17
**Parametric linkage**	**Penetrance**	0.66	0.53	0.80	0.73	0.64	0.61
	**Phenocopy**	0.14	0.14	0.11	0.20	0.00	0.11
	**p-value** **lod score**	0.1150.54	0.2520.28	0.0161.27	0.1710.41	0.0031.87	0.0610.76
**Non-Parametric linkage**	**p-value** **lod score**	0.0650.74	0.0530.81	0.0041.80	0.0151.27	0.0021.99	0.0171.23

NB: Non-affected individuals only provide linkage information and are hence considered if their parent is heterozygous for the studied haplotype, explain why their numbers vary depending on the considered locus.

Having identified the most promising regions, we computed local p-values/lod scores, yet this time including information from the 12 unaffected relatives in addition to the nine cases plus individual II.5. Within each of the six regions we identified the chromosomal position (determined by the location of cross-overs in gametes transmitted to non-affected individuals – non-affected individuals may have inherited part of the segments shared IBD by the cases) that yielded the lowest p-values/highest lod scores (Table 1). Threshold p-values/lod scores to declare significant (expected ones per twenty genome scans) and suggestive linkage (expected ones per genome scan) were empirically determined to be 1.5×10^−4^ (z_sig_ = 3.1) and 1.4×10^−3^ (z_sugg_ = 2.2), respectively (see Methods), very similar to the z_sig_ = 3.3 and z_sugg_ = 1.9 thresholds recommended by Lander & Kruglyak (1995). Thus, none of the six regions exceeded the significance threshold, but two (HSA8 and HSA18) approached suggestive evidence for linkage (Table 1).

We were struck by the fact that for five of the six loci identified on the basis of haplotype sharing amongst cases/obligate carrier, the risk haplotype was under-transmitted by carrier parents to unaffected offspring (Table 1). When pooling data across the six loci, the risk haplotype was transmitted 20 times and non-transmitted 36 times (p = 0.03). This observation suggested that several of the identified loci might jointly influence disease outcome. One testable prediction of this oligogenic model is that the probability of transmission of risk haplotypes from the different loci to unaffected offspring will not be independent: there will be fewer unaffected individuals having inherited the risk haplotype at multiple loci than expected by chance alone. From the 12 unaffected offspring (not including II.5), none had inherited more than three risk haplotypes, three had inherited three risk haplotypes, three two risk haplotypes, five one risk haplotype and one none (Fig. 1). We performed a permutation test to verify whether unaffected individuals with more than three risk haplotypes or with three or more risk haplotypes were significantly underrepresented. For each of the six putative risk loci, we randomly permuted the transmitted haplotype (risk or not) amongst unaffected individuals (hence conditioning on the observed segregation distortion), and counted the proportion of permutations characterized by individuals with more than three risk haplotypes, or by more than three individuals with three or more risk haplotypes. Unaffected individuals with six risk haplotypes were observed in 0.3% of permutations, with five or more risk haplotypes in 8.3% of permutations, with four or more risk haplotypes in 51% of permutations. Permutations with more than three individuals with three or more risk haplotypes were observed in 58% of permutations. Thus this analysis did not yield evidence for a statistically significant under-representation of unaffected individuals with ≥ 3 risk haplotypes (p  =  0.42).

## Discussion

The unusually high incidence as well as the clinical homogeneity of strabismus in the analyzed pedigree suggested a simple genetic determinism. The conducted genome scan essentially excluded the only hypothesis that could reasonably be tested in this single family, i.e. the segregation of a single, autosomal dominant, highly penetrant IBD mutation. Our results are reminiscent of previous reports in large families with multiple affected individuals [4], and add to the evidence of a complex determinism of non-syndromic, concomitant strabismus. We cannot exclude the possibility that two distinct, highly penetrant mutations are underlying strabismus in the two branches of the pedigree (i.e. descendants of individual II.1 versus descendants of individual II.5), which might explain the fact that II.5 is not affected. To the best of our knowledge, however, strabismus is not reported in close relatives of individual II.6. The pedigree was too small to directly test this hypothesis in a meaningful way.

We nevertheless show that two genomic regions come close to reach statistical evidence for suggestive, mapping respectively to 18p11–12 and 8q21. The 18p11–12 signal corresponds to the only region of the genome for which the nine affected individuals and the obligate carrier (II.5) share a clear IBD haplotype. The shared segment spans 18.3Mb, a size which is consistent with expectation (20cM) for a chromosome segment encompassing a hypothetical causative mutation shared by the nine cases and the obligate carrier (II.5). The recombination events defining the boundaries of that segment occurred independently in gametes transmitted to individuals IV.11 and IV.22, respectively. However, the same haplotype was transmitted by carrier parents to five of 13 unaffected offspring, implying a penetrance of only 0.64. We note that the region maximizing the penetrance (10.667.992–11.035.805) contains microsatellite marker D18S464 yielding a non-parametric lod score of 1.34 in the affected sib-pair genome scan conducted by Fujiwara et al. [5]. Interestingly, the corresponding region contains a single large gene coding for the PIEZO2 protein, which plays a role in adapting mechanically activated (MA) currents in somatosensory neurons [8].

The 8q21.2-3 region encompasses a haplotype shared by all but one affected individual (IV.26), implying a phenocopy rate of 0.11, and transmitted only to one of nine unaffected offspring of carrier parents (III.1), implying a penetrance of 0.80. However, the haplotype shared by affected individuals spans only 3.2 Mb which is nearly outside the 95% CI of the size expected for a segment encompassing a causative mutation shared by nine of the ten cases in the considered family. Moreover, the recombinational events defining the boundaries of the shared haplotype occurred in gametes transmitted by the unidentified carrier grand-grand-parent to grand-fathers II.1 and II.5 (either a double recombination event in one of the gametes or two single recombinational events). An alternative explanation would be that the haplotypes inherited by II.2 and II.5 derive from distinct homologues in generation I that happen to share a track of extended identity in the region, a phenomenon which is know to occur at appreciable frequency even for distantly related chromosomes [9]. If true, this would considerably weaken the evidence implicating this region in the determinism of strabismus in this family. We note that Shaaban et al. [6] report suggestive evidence for linkage (z ≤ 2.8) under a dominant model at location 8q24.21. However, microsatellite marker D8S284 adjacent to the linkage peak is located at ∼42 Mb from the interval defined in this study making it unlikely that both signals share a common determination. The 3.2 Mb interval encompasses 18 annotated genes, but no obvious candidate.

Five of the six haplotypes that were identified on the basis of their over-transmission to affected individuals, tended to be under-transmitted to non-affected individuals. This is not expected if at most one of the six identified loci is real (as expected under a monogenic model). Hence, this suggested that the genetic determinism of strabismus might be oligogenic in this pedigree, simultaneously involving several of the identified loci. An independent prediction of this model is that the distribution of the number of risk haplotypes inherited by non-affected individuals should be shifted towards lower values, above what is expected as a result of the under-transmission of the risk haplotypes to non-affected individuals. We have applied a permutation test to evaluate this hypothesis, yet have not found a significant deviation from expectation. It is noteworthy, however, that the power to detect such a shift if it existed was low given the limited sample size available.

The identification of extended haplotypes shared between or – as in this work - within families as a means to map disease-causing mutations has been explored and utilized by others [10]–[13]. This way of analyzing pedigree data is becoming increasingly relevant as dense SNP arrays are becoming the genotyping method of choice. It is paradoxical however that – as information content increases – the computational extraction of a more obvious signal becomes harder. The approach described in this work is clearly “*ad hoc*” and hence not fully satisfactory. Apart from the fact that the analyses were largely performed “manually”, one of the shortcomings is the fact that the utilized “haplotype sharing score”, is not interpretable statistically *per se*. Evaluating the statistical significance of the observed sharing requires subsequent parametric and/or non-parametric analyses focusing on the identified regions. Another drawback is that the method as implemented is likely to be highly sensitive to genotyping errors. Genotyping errors will artifactually interrupt shared segments, potentially causing severe drops in the sharing scores. Finally, by allowing at most one phenocopy when searching for genome segments shared by cases we may have overlooked loci that might ultimately have yielded higher lod scores than the six loci reported in Table 1. Thus, we cannot totally exclude that a linked locus might have been missed in this study as a result of one or more of these issues. There thus appears to be a need for the development of methods that take better advantage of high-density SNP genotypes for linkage analysis in complex pedigrees.

Despite these limitations, our findings are generally consistent with previous genome scans aimed at mapping loci that might underlie the high incidence of concomitant strabismus in large pedigrees available [4]–[7]: there is very little if any evidence that familial forms of concomitant strabismus are determined by individually rare, highly penetrant mutations. Alternative explanations for the observed familial clustering are oligogenic and polygenic models. The oligogenic model assumes more than one, yet still a small number of loci that interact in such a way as to generate highly penetrant genotype combinations. It might be worthwhile to pool the data of multiple extended pedigrees available around the world to test such oligogenic models. The polygenic model, on the other hand, assumes that affected individuals carry common risk alleles with individually small effects at many loci. The addition of multiple such small effects results in a liability that exceeds a threshold value, thereby causing disease. Under this scenario, familial clustering would reflect the unusual concentration of many risk alleles in given families. Detection of the corresponding polygenes could be achieved by performing genome-wide association studies (GWAS) using high-density SNP arrays in large case-control cohorts, and such studies are likely to be underway.

## Methods

### Ethical approval

The research proposal and experimental plan was approved by the Comité d’Ethique Hospitalo-Universitaire de Liège under number B707201317854. It included an informed consent form that was approved and signed by all participants.

### DNA preparation and SNP genotyping

Saliva samples were collected using Oragene DNA (OG250) kits and genomic DNA extracted following the recommendations of the manufacturer. 200 ng of genomic DNA were used for SNP genotyping on HumanCNV370-Quad HD Infinium arrays using standard procedures.

### Confirming sample identity and familial relationships

For all possible pairs of individuals we computed the proportion of autosomal SNPs with genotypes that would not be compatible with a parent-offspring relationship. The outcome was clearly bimodal. All comparisons with low exclusion rates corresponded to expected parent-offspring pairs. Remaining conflicting genotypes were attributed to genotyping errors and were ignored in subsequent haplotype-sharing analyses. The average rate of identity-by-state (IBS) across all SNPs for individuals II.1 and II.5 was within the range of rate of IBS observed for confirmed sibs within the pedigree, confirming that they are brothers.

### Scanning the genome for haplotypes shared by cases

We searched for haplotypes shared by nine or eight cases and the obligate carrier (II.5) using the ASSDOM software that aims at detecting shared dominant haplotypes in a case-control setting [14]. To increase detection power, we first phased as many SNPs as possible using Mendelian rules, i.e. we assigned alleles to the paternal or maternal chromosome based on the genotypes of their parents. For individuals II.1 and II.5 (obligate carrier), phasing is only possible for the homozygous SNPs, as the deceased grand-grand-parents (generation I) were not genotyped. Thus for II.1 and II.5, the two homologous chromosomes have identical “informative” genotype. We then searched for chromosome segments for which the “affected” chromosomes, i.e. the chromosome predicted to carry the IBD dominant mutation (maternal for some, paternal for others; Fig. 1), might share the same haplotype. Such segments are flanked by excluded SNP positions for which affected chromosomes carry distinct alleles, say A vs B. Non-excluded segments were assigned a score which in this case simply reduced to 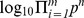 where *m* corresponds to the number of SNPs defining the segment, *p* is the population frequency of the shared allele at SNP *i*, and *n* is the number of informative cases (i.e. with phased genotype at the corresponding SNP). Allelic frequencies, *p*, were estimated from the available non-affected “control” chromosomes. To identify segments shared by the obligate carrier (II.5) plus eight of the nine affected individuals we performed the corresponding analysis five times separately, excluding respectively individuals III.9, IV.5, IV.22, IV.23, IV.26. The location score at a given map position (reported in Fig. 2), corresponded to the best score obtained across the five analyses. Recombination breakpoints (i.e. haplotype boundaries) were identified by manual examination of the SNP genotypes.

### Estimating the expected number of haplotypes shared by cases

**Figure 3:**
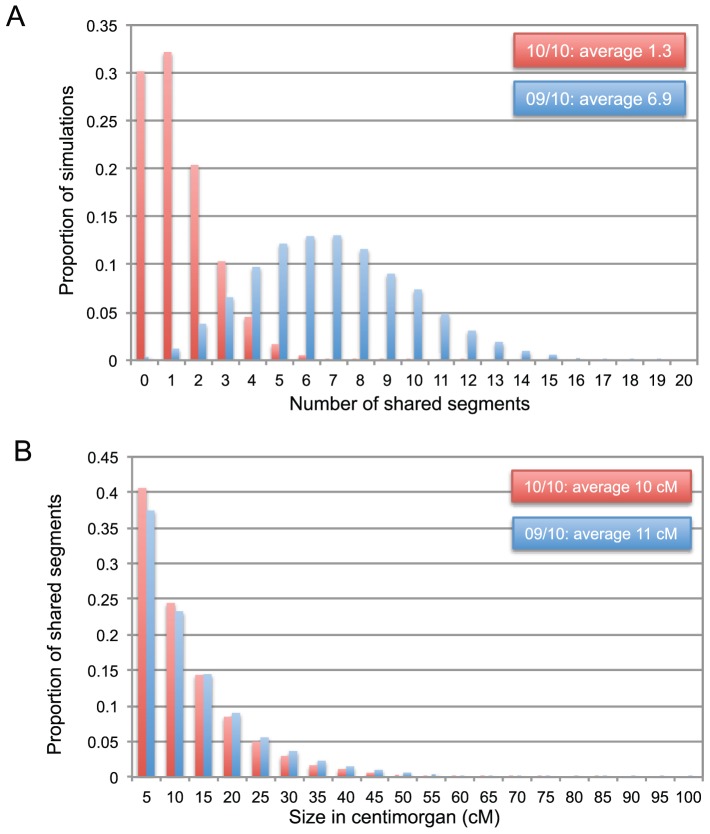
Expected frequency distribution of number and size (in cM) of genomic segments shared IBD by the obligate carrier and affected individuals. (A) Distribution of the expected number of shared segments. (B) Distribution of the expected size of shared segments. (Red) Expected sharing between the obligate carrier and the nine affected individuals. (Blue) Expected sharing between the obligate carrier and eight of the nine affected individuals (blue). Frequency distributions were obtained from 10,000 simulated segregations of 22 autosomes with genetic size according to Kong et al. [15].

We simulated the random segregation of the four grand-grand-paternal genomes in the studied pedigree and particularly to the nine affected individuals and single obligate carrier. The genome was assumed to comprise 22 autosomes with genetic lengths according to [15]. The genome was subdivided in one cM, independently recombining blocks, i.e. we ignored interference. Marker information was assumed to be perfect, i.e. the four grand-grand-parental chromosomes could be unambiguously distinguished from each other and from all other chromosomes in the population. We performed 10,000 simulations and computed the number of segments shared IBD (i.e. tracing back to one of the four grand-grand-parental homologues) by the obligate carrier and either the nine or eight of the nine affected pedigree members. This yielded an average of 1.3 segments shared IBD by the ten individuals, and 6.9 segments shared IBD by nine of the ten individuals. 95% confidence intervals were determined from the frequency distribution of simulations yielding 0, 1, 2... shared segments (Fig. 3A).

### Estimating the expected size of haplotypes shared by cases

The size of the haplotype shared by the *n* selected individuals is defined by the closest cross-over (CO) events that occur on the proximal and distal side of the causative mutation out of *n* meioses. We denote the distance (in Morgan) separating the causative mutation and the nearest out of *n* proximal CO event as 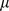. 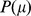, i.e. the density function of 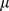, can be derived from the realization that:
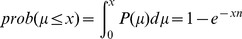



Hence
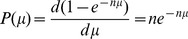



and
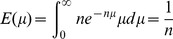



The expected distance between the causal mutation and the closest of *n* distal recombination events will likewise be 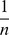. As a consequence, the expected size of the shared segment will be 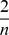.

Simulations perfectly agreed with theoretical predictions when accounting for the fact that the expected size of a haplotype that is shared fortuitously is halve that of a haplotype that is shared because it carries a causative mutation (Fig. 3B). 95% confidence intervals for the size of the shared haplotype were deduced from the simulations.

### Computing parametric lod scores using all individuals in the pedigree

To compute parametric lod scores at a given map position we calculated the likelihood of the data assuming that the locus determined phenotype (H1), as well as the likelihood of the data assuming that the locus did not influence phenotype (H0). For both hypotheses, the likelihood of the data, *L*, was computed as:
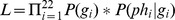



In this, *i* corresponds to one of the 22 genotyped members of the pedigree tracing back to grand-grand-parents I.2 and I.3. *g_i_* is the genotype with respect to the putative risk haplotype (*h*) of interest (identified with ASSDOM as described before), and is therefore either *h+* or ++. *P*(*g_i_*), the probability of *g_i_*, is therefore 0.5 for offspring of parents carrying the index haplotype (equal chance to be *h+* or ++ if parent is *h+*), and 1 for offspring of parents that do not (genotype ++). Under H_1_,
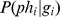
is the penetrance (*Pen*) for affected individuals carrying the haplotype (*h+*), (*1-Pen*) for non-affected individuals carrying the haplotype (*h+*), the phenocopy rate (*Phe*) for affected individuals without the haplotype (++), and (*1-Phe*) for non-affected individuals without the haplotype (++). Maximum likelihood values for *Pen* and *Phe* were directly counted from the data. Under H_0_, 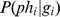
is the prevalence (*Pre*) of strabismus amongst genotyped relatives in the pedigree (i.e. 9/22), and (*1-Pre*) for unaffected individuals (irrespective of their genotype). The lod score, *z*, was computed as 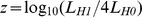. The “4” in the equation reflects the fact that the 22 related pedigree members can share any of the four grand-grand-paternal haplotypes by chance (H0). To determine corresponding nominal *p*-values, we considered that 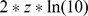 has a chi-squared distribution with one degree of freedom under H0. The corresponding lod score is parametric because it is determined by *Pen, Phe* and *Pre*, the parameters defining the genetic model.

### Computing non-parametric lod scores using all individuals in the pedigree

This was achieved by determining the probability of the fortuitous association between risk haplotype (identified with ASSDOM as described above) and disease status, given the structure of the examined pedigree. To that end, we performed 10×10^6^ *in silico* “droppings” of a hypothetical mutation in the real genealogy, and classified the outcome of each dropping by (i) the number of informative affected individuals with the mutation (cases-T in Table 1), (ii) the number of informative affected individuals without the mutation (cases-NT in Table 1), (iii) the number of informative non-affected individuals with the mutation (non-affected-T in Table 1), and (iv) the number of informative non-affected individuals without the mutation (non-affected-NT in Table 1). By informative, we mean that their parent had to be heterozygous for the hypothetical mutation. We observed a total of 737 distinct segregation patterns including the six reported in Table 1. We then sorted the corresponding segregation patterns by their frequency of occurrence. The statistical significance of a given pattern was then computed as the sum of the frequencies of all patterns that were as rare or rarer than the observed one. This approach is similar in principle to Fisher’s exact test. To facilitate comparison with the literature and the parametric lod scores, we determined the value of *z*, such that 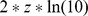 would yield the corresponding *p*-value when considered a chi-squared variable with one degree of freedom.

### Determining genome-wide significant and suggestive lod score thresholds

Following Lander & Kruglyak [16], significant thresholds are expected to be exceeded by chance alone once every twenty genome-scans, while suggestive thresholds are expected to be exceeded on average once per genome scan. Thus the genome-wide (i.e. accounting for the realization of multiple tests) *p*-value of a significant lod score is ≤ 0.05 and of a suggestive lod score is ≤ 0.37. To see the latter, a lod score that is not exceeded in 37% of genome scans is exceeded on average once per genome scan, as 0.37 = e^−1^ (assuming that such events are Poisson distributed). We know the nominal p-value of lod score values, as 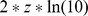 is assumed to asymptotically have a chi-squared distribution with one degree of freedom under H_0_ (see above). To determine the corresponding genome-wide p-value requires knowledge of the number of independent tests performed when scanning the genome. The above-mentioned simulations provide that estimate. Indeed, the probability that the nine cases + II.5 fortuitously share one of the four grand-grand-paternal alleles IBD at a specific locus is 4/2^10^  =  1/256. The fact that the nine cases + II.5 share on average 1.3 grand-grand-parental alleles IBD anywhere in the genome tells us that the genome segregates as 256*1.3 = 333 independent segments. Thus, significant and suggestive nominal p-values are ∼0.05/333 = 0.00015 and ∼0.37/333 = 0.0011, corresponding to lod scores of 3.12 and 2.31, respectively. Note that these thresholds are very similar to those suggested by Lander & Kruglyak [16] for “lod score analysis in human”, namely 3.3 and 1.9, respectively.

## References

[1] Engle EC (2007) Genetic basis of congenital strabismus. Arch Ophthalmol 125: 189–193. 17296894 10.1001/archopht.125.2.189

[2] Miyake N, Chilton J, Psatha M, Cheng L, Andrews C, et al (2008) Human CHN1 mutations hyperactivate alpha-2-chimaerin and cause Duane's retraction syndrome. Science 321: 839–843. 18653847 10.1126/science.1156121 PMC2593867

[3] Tischfield MA, Baris HN, Wu C, Rudolph G, Van Maldergem L, et al (2010) Human TUBB3 mutations perturb microtubule dynamics, kinesin interactions, and axon guidance. Cell 140: 74–87. 20074521 10.1016/j.cell.2009.12.011 PMC3164117

[4] ParikhV, Shugart YY, DohenyKF ZhangJ, Li L, et al (2003) A strabismus susceptibility locus on chromosome 7p. Proc. Nat. Acad. Sci. 100: 12283–12288. 10.1073/pnas.2035118100 PMC218750 14519848

[5] Fujiwara H, Matsuo T, Sato M, Yamane T, Kitada M, et al (2003) Genome-wide search for strabismus susceptibility loci. Acta Medica Okayama 57: 109–116. 12908008 10.18926/AMO/32833

[6] Shaaban S, Matsuo T, Fujiwara H, Itoshima E, Furuse T, et al (2009) Chromosomes 4q28.3 and 7q31.2 as new susceptibility loci for comitant strabismus. Invest Ophthalmol Vis Sci. 50: 654–661. 10.1167/iovs.08-2437 18824738

[7] Rice A, Nsengimana J, Simmons IG, Toomes C, Hoole J, et al (2009) Replication of the recessive STBMS1 locus but with dominant inheritance. Invest Ophthalmol Vis Sci 50: 3210–3217. 19218600 10.1167/iovs.07-1631

[8] Coste B, Mathur J, Schmidt M, Earley TJ, Ranade S, et al (2010) Piezo1 and Piezo2 are essential components of distinct mechanically activated cation channels. Science 330: 55–60. 20813920 10.1126/science.1193270 PMC3062430

[9] International HapMap Consortium (2007) A second generation human haplotype map of over 3.1 million SNPs. Nature 449: 851–861. 17943122 10.1038/nature06258 PMC2689609

[10] Van der Meulen (1997) MA (1997) Haplotype sharing analysis in affected individuals from nuclear families with at least one affected offspring. Genetic Epidemiology 14: 915–919. 9433600 10.1002/(SICI)1098-2272(1997)14:6<915::AID-GEPI59>3.0.CO;2-P

[11] Bourgain C, Génin E, Holopainen P, Mustalahti K, Mäki M, et al (2001) Use of closely related affected individuals for the genetic study of complex diseases in founder populations. Am J Hum Genet 68: 154–159. 11102286 10.1086/316933 PMC1234909

[12] Beckmann L (2005) Haplotype sharing analysis using mantel statistics. Hum Hered 59: 67–78. 15838176 10.1159/000085221

[13] Thomas A, Camp NJ, Farnham JM, Allen-Brady K, Cannon-Albright LA (2008) Shared genomic segment analysis. Mapping disease predisposition genes in extended pedigrees using SNP genotype assays. Ann Hum Genet 72: 279–287. 18093282 10.1111/j.1469-1809.2007.00406.x PMC2964273

[14] Durkin K, Coppieters W, Drögemüller C, Ahariz N, Cambisano N, et al (2012) Serial translocation by means of circular intermediates underlies colour sidedness in cattle. Nature 482: 81–84. 22297974 10.1038/nature10757

[15] Kong A, Gudbjartsson DF, Sainz J, Jonsdottir GM, Gudjonsson SA, et al (2002) A high-resolution recombination map of the human genome. Nat Genet. 31: 241–247. 10.1038/ng917 12053178

[16] Lander E, Kruglyak L (1995) Genetic dissection of complex traits: guidelines for interpreting and reporting linkage results. Nat Genet. 11: 241–247. 10.1038/ng1195-241 7581446

